# An Encoding Technique for Multiobjective Evolutionary Algorithms Applied to Power Distribution System Reconfiguration

**DOI:** 10.1155/2014/506769

**Published:** 2014-10-23

**Authors:** J. L. Guardado, F. Rivas-Davalos, J. Torres, S. Maximov, E. Melgoza

**Affiliations:** ^1^Instituto Tecnológico de Morelia, Avenida Tecnológico 1500, 58120 Morelia, MICH, Mexico; ^2^Instituto Tecnológico Superior de Irapuato, Carretera Irapuato, Silao Km. 12.5, 36821 Irapuato, GTO, Mexico

## Abstract

Network reconfiguration is an alternative to reduce power losses and optimize the operation of power distribution systems. In this paper, an encoding scheme for evolutionary algorithms is proposed in order to search efficiently for the Pareto-optimal solutions during the reconfiguration of power distribution systems considering multiobjective optimization. The encoding scheme is based on the edge window decoder (EWD) technique, which was embedded in the Strength Pareto Evolutionary Algorithm 2 (SPEA2) and the Nondominated Sorting Genetic Algorithm II (NSGA-II). The effectiveness of the encoding scheme was proved by solving a test problem for which the true Pareto-optimal solutions are known in advance. In order to prove the practicability of the encoding scheme, a real distribution system was used to find the near Pareto-optimal solutions for different objective functions to optimize.

## 1. Introduction

Modern societies require a complex system of generating plants, interconnected transmission lines, and distribution systems. The overall power losses in the generation, transmission, and distribution of electrical energy are estimated in 8–15% [1]. These figures mean that there is still room for reducing losses in electrical power system.

An alternative to reduce power losses in distribution systems is network reconfiguration [2]. However, this is one of the most computationally demanding problems in distribution systems because it requires the optimization of several objective functions related to the operational efficiency of distribution systems such as power losses, voltage deviations, circuit breaker operations, and expected energy not supplied, among others, while all network constraints are satisfied, for example, line currents and voltage drop limits and a radial configuration.

Considering that modern distribution system may have thousands of possible topologies and the nonlinear nature of power losses, the distribution system reconfiguration (DSR) problem can be defined as a highly complex, combinatorial, and nondifferentiable optimization problem. Furthermore, the radiality constraint introduces additional complexity to the problem, especially in large size distribution networks. Because of this, new algorithms are emerging continuously to deal with the complexity of optimizing radial power distribution system operation.

Metaheuristic algorithms using a multiobjective approach for solving the DSR problem have been very popular in the last decade [3–8], and a literature review is given in [9]. In the multiobjective approach, more than one objective function is optimized simultaneously, such as minimizing power losses and voltage deviations in the system, balancing loads in transformers, minimizing the number of operated switches during the DSR process, and maximizing system reliability. In practice, some of these objective functions are conflicting between each other and it is not possible to find a single solution that simultaneously optimizes all the objective functions, but there exists the alternative of obtaining a set of solutions, known as the Pareto-optimal solutions, which represents a tradeoff between all the conflicting objectives. Evolutionary algorithms (EAs) have been used extensively for solving multiobjective optimization problems in several areas [10, 11] and in particular obtaining the Pareto-optimal solutions for DSR problems [12].

One of the main challenges in EA design for loss reduction in DSR problems is how to encode the possible solutions or system topologies in order to make the search efficient and effective. A good encoding strategy should be capable of representing all possible solutions, must facilitate that genetic operators are being implemented in an efficient way, and also should be inexpensive in evaluating the fitness function and constraints while moving easily between the encoded solution and its representation. In addition, the encoding strategy must generate only solutions with radial topologies; otherwise an excessive number of unfeasible solutions may be generated, reducing the efficiency and effectiveness of the search process. According to research results presented in [13], less than 1% of all solutions are feasible in the solutions space.

The problem of finding the Pareto-optimal reconfigurations in distribution networks considering several objective functions is quite similar to the multiobjective minimum spanning tree (MO-MST) problem. This analogy is relevant because, in [14, 15], comparisons of several encoding schemes for solving MO-MST problems are presented. From these comparisons it can be said that some encoding schemes perform better than others and that the effectiveness of the encoding scheme affects the quality of the Pareto-optimal solutions obtained. As far as the authors know, the Edge Window Decoder (EWD) technique [16] has not been applied for solving the MO-MST and DSR problems.

In this paper, an encoding scheme for representing the system configuration during DSR problems is presented. The proposed encoding scheme was embedded in the NSGA-II [17] and SPEA2 algorithms [18]; however, the encoding scheme can be easily adapted to many other multiobjective evolutionary algorithms. The encoding technique was tested in a MO-MST problem for which the true Pareto-optimal solutions are known in advance. In order to prove the practicability of the encoding scheme, a real distribution system was used to find the near Pareto-optimal solutions for different scenarios. This work also demonstrates that the proposed encoding technique can be used successfully for solving DSR problems in a multiobjective formulation and not only for monoobjective optimization problems [19].

The main contributions of the paper are as follows: (1) the encoding technique proposed, combined with specialized genetic operators, can explore the search space efficiently, finding the true Pareto-optimal solutions for MO-MST problems and near Pareto-optimal solutions for DSR problems; (2) the proposed encoding techniques and genetic operators are capable of dealing with the radiality constraint in a multiobjective search space; (3) the encoding technique enables the search process to find well-dispersed near Pareto-optimal solutions in large-scale power distributions systems.

## 2. Encoding Technique Description 

### 2.1. Problem Statement

The DSR problem basically consists in determining a new topology that minimizes different objective functions. Since the main concern during normal distribution system operation is efficiency and power quality, in this paper three objectives are considered: minimizing power losses, minimizing bus voltage deviations, and minimizing the number of operated switches during the DSR process. The objective function for loss reduction due to Joule effect in the different line sections is

(1) $$\begin{array}{c} \min f_{1}=\min\left(P_{\text{LOSS}}=\overset{}{\underset{i\in N_{i}}{\sum }}3I_{i}^{2}R_{i}\right), \end{array}$$

where *P*_LOSS_ are the total power losses in the system, *I*_*i*_ is the current through section  *I*_*i*_, *R*_*i*_ is the resistance of line section  *i*, and *N*_*i*_ is the total number of line sections contained in the system.

The objective function to optimize voltage deviations at every node in the distribution system is

(2) $$\begin{array}{c} \min f_{2}=\min\left\{\sqrt{\frac{\sum_{i=1}^{nb}{\left(V_{i}-1\right)}^{2}}{nb\left(nb-1\right)}}\right\}, \end{array}$$

where *nb* is the total number of nodes in the system and *V*_*i*_ is the voltage at the *i*th node. Minimizing *f*_2_ means that voltage levels at every system node are closer to 1.0 pu.

Finally, it is also desirable that during the reconfiguration process the number of operated switches is as small as possible in order to reduce the reconfiguration time, the probability of human error, and operation costs. The objective function used to minimize the number of operated switches in the reconfiguration process is

(3) $$\begin{array}{c} \min f_{3}=\overset{N_{s}}{\underset{i=1}{\sum }}\left|S_{i}-S_{0i}\right|, \end{array}$$

where *N*_*s*_ is the total number of switches in the system, *S*_0*i*_ and *S*_*i*_ are the status of switch *i* in the original and new distribution system configuration, respectively, and *f*_3_ indicates the number of switches that have modified its status after system reconfiguration.

All new topologies generated during the DSR process must also comply with the following system constraints: (a) a radial network structure must be obtained after network reconfiguration in which all loads are energized, (b) the apparent power on each line section must be smaller than the maximum apparent power allowed, (c) the voltage level in a given node must be within allowed limits, and (d) the apparent power at the substations transformers must be within allowed limits. These constraints can be formally expressed as follows:

(4) $$\begin{array}{c} S_{i}\leq S_{i\max},\\ V_{i\min}\leq V_{i}\leq V_{i\max},\\ S_{Ai}\leq {S_{A}}_{i\max}, \end{array}$$

where *S*_*i*_ and *S*_*i*max⁡_ are the apparent power and maximum capacity limit of line section  *i*, *V*_*i*_ is the voltage magnitude of bus *i*, *V*_*i*min⁡_ and *V*_*i*max⁡_ are the minimum and maximum voltage limits at bus *i*, and *S*_*Ai*_ and *S*_*A*__*i*max⁡_ are the apparent power and maximum capacity limit of substation transformer *i*.

In addition, the multiobjective optimization process for solving the DSR problem has two goals that must be fulfilled [20]: (a) to find a set of solutions as close as possible to the true Pareto-optimal solutions and (b) to find a set of solutions as diverse as possible.

### 2.2. The Proposed Multiobjective Evolutionary Algorithm

The proposed encoding scheme based on the EWD technique, but modified and adapted to DSR problems, was implemented in two multiobjective EAs: the Strength Pareto Evolutionary Algorithm 2 (SPEA2) and Nondominated Sorting Genetic Algorithm II (NSGA-II). Also, specialized recombination and mutation operators were developed in order to guarantee a radial configuration in all the new possible solutions generated along the evolutionary process. The salient steps of the developed encoding scheme and the evolutionary operators for the DSR problem are described in the next sections.

### 2.3. The Encoding Scheme

**Figure 1:**
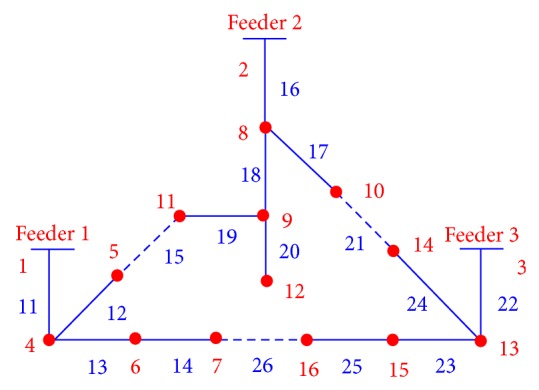
Power distribution system for optimal reconfiguration.

The EWD encoding scheme was used to codify each possible solution in the DSR problem. In order to describe the encoding scheme, let us consider the distribution network shown in Figure 1, which has 13 line sections with switches normally closed, three line sections with normally open switches, and 13 power load nodes. In order to manipulate the system shown in Figure 1 as a spanning tree, the nodes referring to the power substations (1), (2), and (3) are considered as a single node. Line sections are in blue color and power load nodes in red.

The encoding process consists basically of the following two steps. First, an initial string is built up by visiting every node and branch in the network without leaving the paper. For example, for the network in Figure 1, a possible string of visited nodes is *S* = (1,4, 5,11,9, 12,9, 8,2, 3,13,14,10,8, 10,14,13,15,16,7, 6,4). In the second step, a moving window of length two reads two nodes in the initial string from left to right, and if the second node has not been already included in the developing edge set ES{}, the edge is included. On the other hand, if the second node in the moving window is already included, this means that either the edge has already been included or a loop has been formed. In the first case, the edge is skipped, and in the second case, a branch is randomly selected and opened to break the loop. Any window containing copies of the same node identifier must be ignored; for example, when “2, 3” in the string is examined by the moving window, it would be passed over without effect, and the next window (starting with the second number in the window) would be now considered. In this example, nodes (1), (2), and (3) are copies because they are considered as a single node.

**Figure 2:**
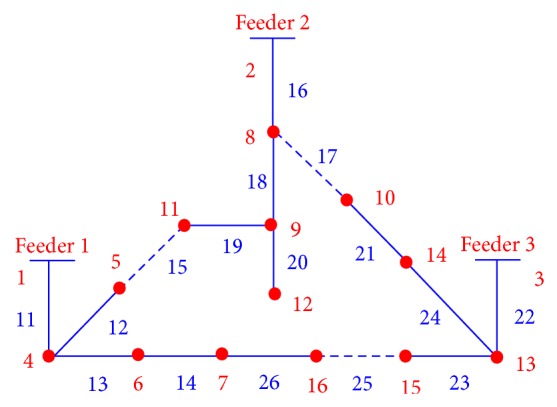
New configuration for the distribution system.

This process is continued through the end of the string. Observe that edge sets (5,11), (14,10), and (16,7) were randomly selected and opened because they form part of a loop. Finally, the selected edge set becomes ES = {(1,4), (4,5), (11,9), (9,12), (9,8), (8,2), (3,13), (13,14), (10,8), (13,15), (15,16), (7,6), (6,4)}. If, instead of (5,11) and (14,10), the edge sets (5,11), (10,8), and (15,16) are randomly opened, then Figure 2 is obtained, which represents a different radial configuration for the distribution network shown in Figure 1.

The edge set ES{} can also be expressed in terms of line sections; for example, for Figure 1, LS = {11,12,19,20,18,16,22,24,17,23,25,14,13}. This encoding procedure assures the radiality for any created configuration. Thus, the initial population is built up from the string *S*, when a loop is formed and then randomly a branch is disconnected from the system. The process is repeated for *N* individuals forming the initial population.

### 2.4. Crossover Operator

**Figure 3:**
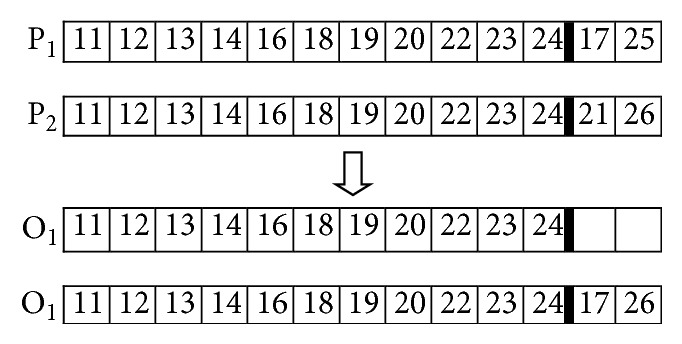
Offspring generated during crossover.

The crossover operator used in this study is presented in [21], and it is implemented in two steps. In the first step, from two parent solutions, a set of line sections common to both solutions are selected in order to initialize the offspring. In the second step, line sections in either parent are randomly and successively selected to be included in the offspring. Only line sections that do not introduce closed loops are included in the offspring. If the offspring is not yet a full spanning tree, other line sections from the candidate line sections and not contained in the parents are selected randomly until a full spanning tree is built up. In order to illustrate the crossover operator let us consider the networks shown in Figure 1 like parent 1 (P1) and the network shown in Figure 2 like parent 2 (P2). The offspring (O1) is shown in Figure 3.

**Figure 4:**
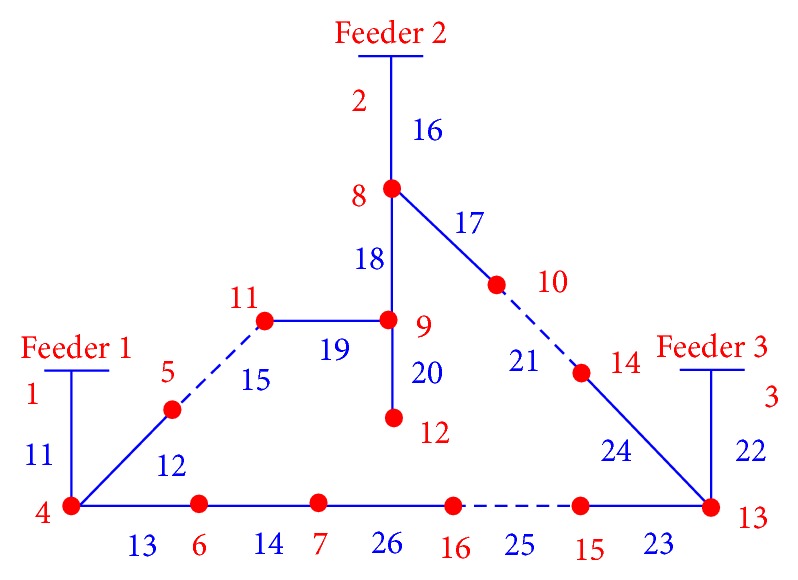
Distribution network after crossover.

This crossover operator generates only legal offspring solutions (radial configuration), avoiding problems of low heritability and topological unfeasibility.  Figure 4 shows the new distribution network configuration after recombination.

### 2.5. Mutation Operator

**Figure 5:**
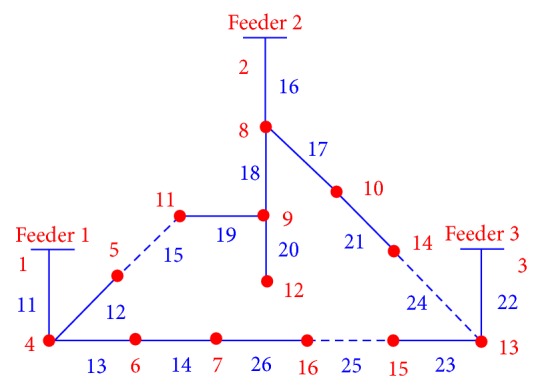
Distribution network after mutation.

Like the crossover operator, the mutation operator implemented in this research was presented in [21], and it is described as follows. In the offspring, Figure 4, a candidate line section currently not connected is randomly selected and connected in the offspring; for example, line section  21 is connected. This action generates a closed loop. Then, randomly, a line section in the loop is selected (excluding the new line section connected) and disconnected from the offspring. In Figure 4, line section 24 is disconnected. The new distribution network obtained after mutation is shown in Figure 5.

## 3. Results and Discussion

The proposed encoding technique was embedded in two of the most representative state-of-the-art multiobjective evolutionary algorithms: the Strength Pareto Evolutionary Algorithm 2 (SPEA2) and the Nondominated Sorting Genetic Algorithm II (NSGA-II). Both algorithms are now applied to solve theoretical and real-world problems.

### 3.1. Assessment of the Encoding Strategy

Evolutionary algorithms for solving single- or multiobjective optimization problems are often criticized for their lack of theoretical foundation. In the case of multiobjective optimization problems a question frequently arises: How close are the obtained solutions to the Pareto-optimal front? In the EA literature such questions are often addressed by first solving a set of test problems for which the optimal solutions are known in advance. Such exercises provide confidence about the efficacy of the proposed procedure before being applied to a real problem where the optimal solutions are not known. Therefore, testing the proposed encoding strategy in solving a given MO-MST problem is appropriate in order to assess how effective the encoding strategy can be for solving multiobjective DSR problems.

The MO-MST problem used to assess the proposed encoded strategy is described as follows [14]: let us consider a complete graph with 10 nodes and 45 lines (every node has a direct connection with the rest of the nodes), and each line has two weights, *w*1 and *w*2, which are nonnegative real numbers and represent attributes, for example, economical cost and a reliability index. The appendix shows the weights *w*1 and *w*2 for each line, respectively. The problem consists in finding the set of Pareto-optimal spanning trees considering two objective functions to minimize: one objective function is the sum of the weights *w*1 and the other one is the sum of the weights *w*2. The true Pareto-optimal solutions to this problem are known in advance and reported in [22].

**Figure 6:**
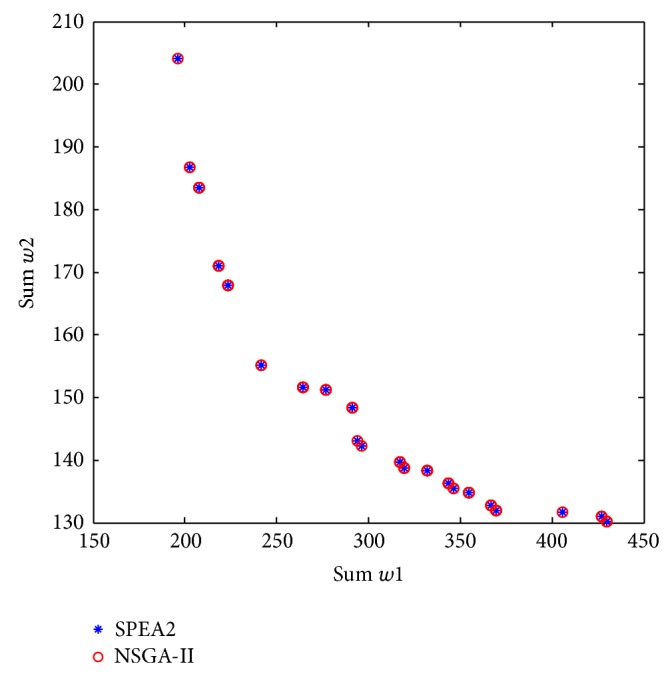
Pareto-optimal front for the multiobjective minimum spanning tree problem.

Figure 6 shows the results obtained for this case of study using the proposed encoding scheme in the SPEA2 and NSGA-II algorithms. Both algorithms found the same Pareto-optimal solutions in a single simulation run. These results demonstrate that the proposed encoding scheme and the evolutionary operators are effective for solving MO-MST problems. The study can also be considered as a validation of the encoding strategy and now can be applied to solve real multiobjective DSR problems.

### 3.2. Solving Multiobjective DSR Problems

A real distribution system with 84 nodes and 531.99 kW of power losses in its initial configuration is used for practical validation purposes. The system has been used by many researchers for comparative purposes and the one line diagram and system data can be found in [23]. Based on a number of experiments, the EA developed used the following parameters in all the cases of study: initial population of 80 individuals, crossing rate of 0.8, and mutation rate of 0.8.

**Case 1 (multiobjective optimization for power losses and operated switches).**

**Figure 7:**
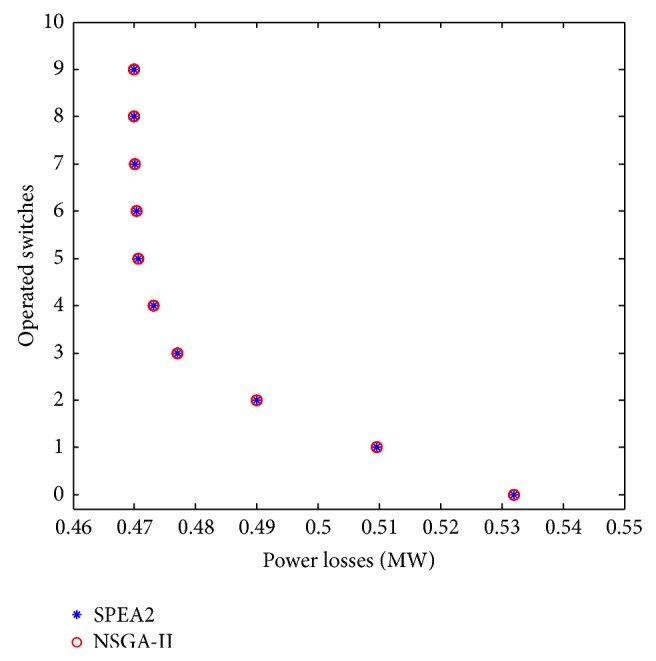
Approximate Pareto-optimal solutions to the problem of optimizing power losses *f*_1_ and operated switches *f*_3_.

In this case, the problem consists in simultaneously minimizing power losses, *f*_1_, and the number of operated switches, *f*_3_. At the end of the multiobjective optimization process, the EAs based on SPEA2 and NSGA-II algorithms must provide a set of solutions which describe a near Pareto-optimal front for the DSR problem. The different solutions obtained are shown in Figure 7.

From Figure 7, it can be observed that both optimization algorithms have practically the same performance and their solutions are superimposed in the same near Pareto-optimal front. Also, it can be seen that relatively well-dispersed solutions allow the distribution system operators to have a better opportunity to select the best solution considering other operational factors.

Table 1 shows some characteristics of the different solutions depicted in Figure 7. For example, solution number 10 has the smallest power losses, 469.9 kW, but in order to reach this topology the status of at least nine switches in the distribution network has to be modified. It should be mentioned that this minimal value of losses has also been obtained using a monoobjective approach in different algorithms like the Plant Growth Simulation Algorithm (PGSA), Simulated Annealing (SA), Ant Colony Search Algorithm (ACSA), and Variable Scaling Hybrid Differential Evolution (VSHDE) [19]. Figure 7 and Table 1 show that, in a multiobjective scenario, the proposed encoding strategy is capable of representing and solving the DSR problem, obtaining a near Pareto-optimal front, and optimizing power losses and the number of operated switches.

**Case 2 (multiobjective optimization for power losses and voltage deviations).**

**Figure 8:**
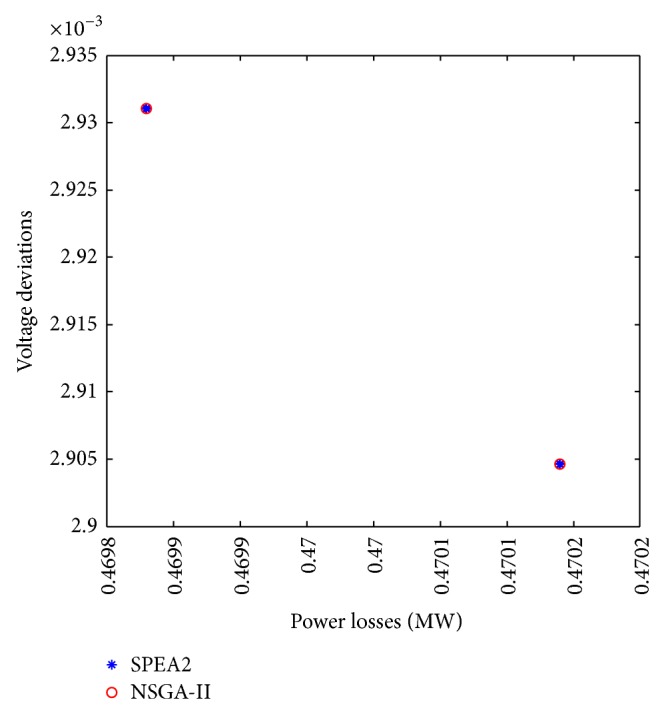
Approximate Pareto-optimal solutions to the problem of optimizing power losses *f*_1_ and voltage deviations *f*_2_.

A second study in the same distribution network was carried out. It is now required to optimize using a multiobjective approach the following objective functions: power losses, *f*_1_, and voltage deviations, *f*_2_, at the different nodes in the distribution network. The near Pareto-optimal solutions obtained by both algorithms are shown in Figure 8. Observe that the Pareto-optimal front for this particular case is constituted by only two solutions.

This behavior is explained by the fact that, for this particular study, during the optimization process improvements in *f*_1_ also improves *f*_2_. In multiobjective optimization this behavior occurs when both objective functions are not contradictory between them, and in terms of electrical network analysis this means that in most of the time any reconfiguration for loss reduction also improves the voltage profile in the network.

**Figure 9:**
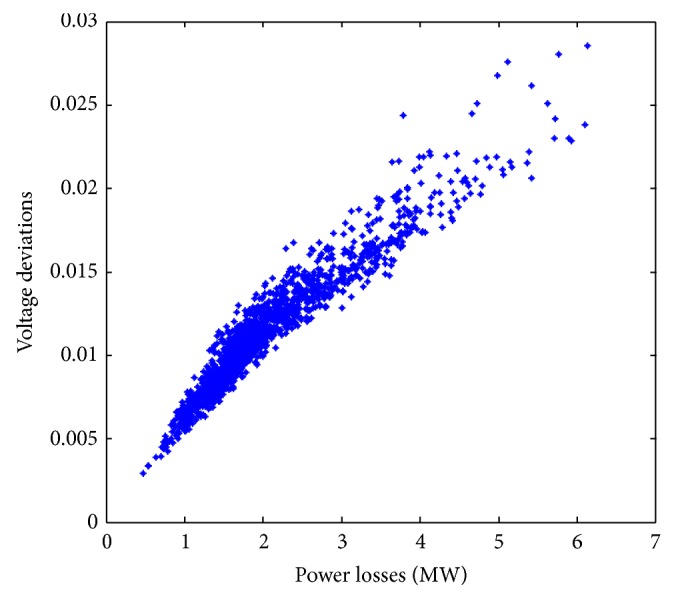
Solutions in the search space for optimizing power losses and voltage deviations.

In this particular case there exist only two configurations for which both objective functions are competing between them and both configurations are nondominated solutions.  Figure 9 shows different solutions in the search space formed by *f*_1_ and *f*_2_ in a larger scale, where solutions with higher losses and voltage deviations are also shown.

Table 2 shows some characteristics of the solutions corresponding to the Pareto-optimal front shown in Figure 8. Observe that the encoding strategy in NSGA-II and SPEA2 reaches the same solutions, which have different power losses and voltage deviations. Again, it should be mentioned that these results are also obtained using a monoobjective approach, which demonstrate that the encoding strategy also works properly and achieves a near Pareto-optimal front with minimal losses and voltage deviations.

**Case 3 (multiobjective optimization for voltage deviations and operated switches).**

**Figure 10:**
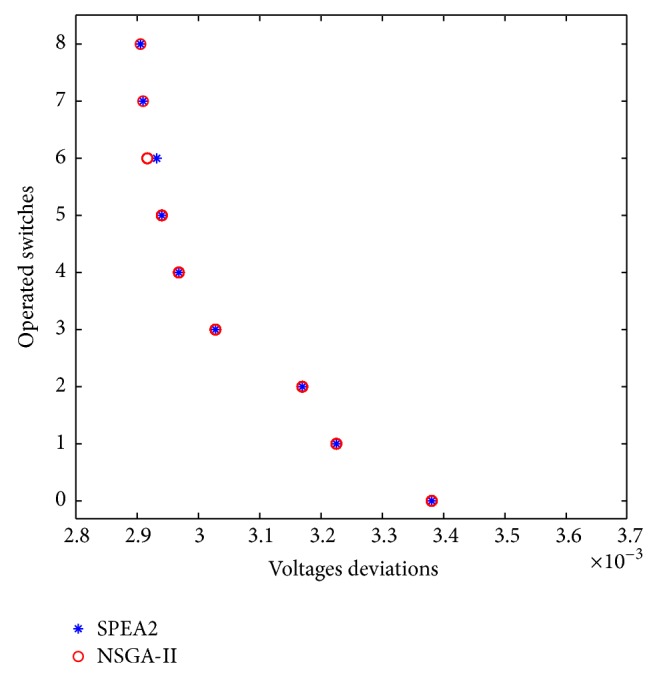
Approximate Pareto-optimal solutions to the problem of optimizing voltage deviations *f*_2_ and the number of operated switches *f*_3_.

Now, the DSR problem is solved optimizing voltage deviations in the different system nodes, *f*_2_, and also the number of operated switches, *f*_3_.  Figure 10 shows the set of solutions obtained at the end of the multiobjective optimization process.

In general, the form of the approximate Pareto-optimal front obtained with NSGA-II and SPEA2 algorithms using the encoding strategy is basically the same except one set of solutions on which both algorithms achieve different values. However, it should be remembered that these are metaheuristics methods, not analytical ones, and there exists the probability of some small difference in the results in each simulation run.

Table 3 shows the characteristics of the different solutions. Observe that the solutions in disagreement differ only in the magnitude of the voltage deviations since the number of operated switches is the same; see solutions 7 and 8. The minimal differences between the near Pareto-optimal front obtained with NSGA-II and SPEA2 are due to the stochastic characteristics of both methods.

**Case 4 (multiobjective optimization for *f*_1_, *f*_2_, and *f*_3_).**

**Figure 11:**
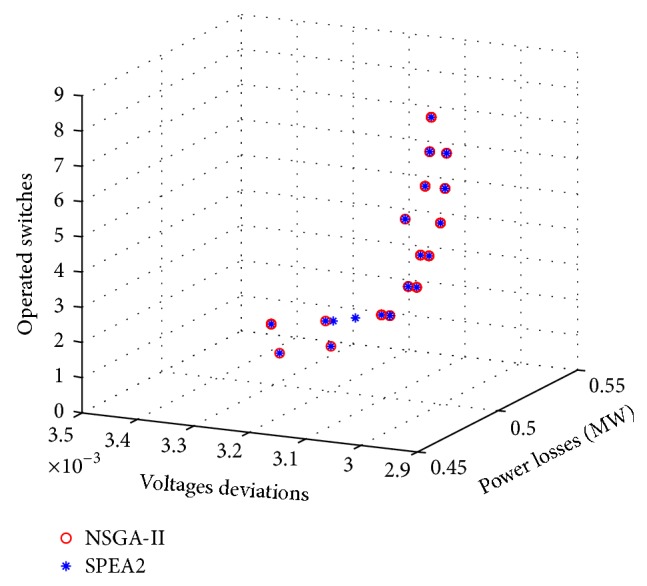
Approximate Pareto-optimal solutions to the problem of optimizing *f*_1_, *f*_2_, and *f*_3_.

In this case it is desired to optimize simultaneously the three objective functions, *f*_1_, *f*_2_, and *f*_3_, in order to minimize losses, voltage deviations, and the number of operated switches. The obtained results for this multiobjective optimization are presented in Figure 11.

A close comparison with previous results shows that this set of solutions contains the best solutions found in Figures 7, 8, and 10. In this sense, the encoding strategy proposed to solve the DSR problem is capable of applying a multiobjective approach to the three functions *f*_1_, *f*_2_, and *f*_3_ and achieve the same results as those obtained using a separate two-function optimization or an individual monoobjective optimization.

Table 4 shows some characteristics of the different solutions. In the great majority of cases the set of solutions are practically superimposed, except in two cases: solutions 7 and 8. Remember that at the end these are probabilistic optimization methods and some small discrepancy between the solutions is understandable. Also the great majority of the solutions were also obtained using a monoobjective or biobjective optimization approach, which demonstrates that the obtained results are coherent.

## 4. Conclusions

One of the main challenges in designing an EA to solve DSR problems is how to encode the possible solutions in order to make the EA process efficient and effective. The demand for solutions with radial topologies makes developing a good encoding strategy for solving the DSR problem more difficult, since this constraint can make encoding methods and their genetic operators generate an excessive number of unfeasible solutions, reducing the efficiency and effectiveness of the search process. Additionally, in a multiobjective optimization context, the effectiveness of an encoding scheme affects the quality of the Pareto-optimal solutions obtained, expressed in terms of a set of solutions as diverse and close as possible to the true Pareto-optimal solutions.

In this paper, an encoding technique for representing the distribution system topology during DSR problems has been presented. The technique is based on the EWD technique but modified and adapted for solving DSR problems. The encoding scheme was embedded in the NSGA-II and SPEA2 algorithms and applied to obtain the true Pareto-optimal solutions in a MO-MST problem. The encoding scheme was also applied to obtain the approximate Pareto-optimal solutions in a real distribution system during system reconfiguration considering a multiobjective approach.

The obtained results show that the proposed encoding technique enables the NSGA-II and SPEA2 algorithms to find the true Pareto-optimal solutions, which means that the proposed encoding technique and their genetic operators are suitable to be used in multiobjective evolutionary algorithms to solve MO-MST based problems. Also, to the best knowledge of the authors, this is the first time that an encoding technique proposed for DSR problems is tested in a MO-MST problem.

In order to prove the practicability of the encoding scheme, a real distribution system was used to find the near Pareto-optimal solutions for different scenarios. The objective functions to optimize used in this analysis are aimed at minimizing power losses, voltage deviation on system nodes, and the number of operated switches during the reconfiguration process. However, any other objective function can be incorporated into the analysis.

According to the results of this analysis, the encoding technique is suitable to find the near Pareto-optimal solutions in different scenarios of DSR. Also, the obtained Pareto-optimal solutions showed a well-dispersed characteristic, and some of these solutions correspond to the optimal solutions presented in the literature for monoobjective formulations used for optimizing a single variable in DSR problems.

From all the obtained results, it can be concluded that the encoding strategy is valid and can be successfully used for solving single-objective optimization problems as well as multiobjective optimization problems. This is relevant because it has been proved that the good performance of some encoding techniques for solving single-objective optimization problems is not necessarily the same, or approximately the same, when they are applied for solving multiobjective optimization problems [14, 15].

In addition, the versatility of the encoding strategy allows for the use of efficient operators for crossover and mutation which guarantees an excellent global and local search, generating only legal solutions. These genetic operators can also be adapted easily to the problem to be solved.

Since the performance of proposed encoding strategy was analyzed in two multiobjective evolutionary algorithms, research should be done on other encoding techniques to be able to draw more general conclusions related to the encoding strategy proposed in this paper. Possible interesting directions would be comparing different encoding techniques tested on distributions networks with different characteristics such as level of power demand, total length of the feeders, and average conductor size of the feeders.

## Figures and Tables

**Table 1:** Characteristics of the approximate Pareto-optimal solutions in Figure 7.

Solution number	Line sections open	Power losses (MW)	Switching operations
1	84, 85, 86, 87, 88, 89, 90, 91, 92, 93, 94, 95, 96	0.5320	0
2	34, 84, 85, 86, 87, 88, 89, 90, 91, 92, 93, 95, 96	0.5096	1
3	7, 34, 84, 86, 87, 88, 89, 90, 91, 92, 93, 95, 96	0.4900	2
4	7, 34, 63, 84, 86, 87, 88, 89, 90, 91, 92, 93, 95	0.4771	3
5	7, 34, 63, 72, 84, 86, 88, 89, 90, 91, 92, 93, 95	0.4731	4
6	7, 34, 63, 72, 83, 84, 86, 88, 89, 90, 92, 93, 95	0.4707	5
7	7, 13, 34, 63, 72, 83, 84, 86, 89, 90, 92, 93, 95	0.4704	6
8	7, 13, 34, 55, 62, 72, 83, 86, 89, 90, 92, 93, 95	0.4701	7
9	7, 13, 34, 39, 55, 62, 72, 83, 86, 89, 90, 92, 95	0.4700	8
10	7, 13, 34, 39, 42, 55, 62, 72, 83, 86, 89, 90, 92	0.4699	9

**Table 2:** Characteristics of the approximate Pareto-optimal solutions in Figure 8.

Solution number	Line sections open	Power losses (Mw)	Voltage deviation
1	7, 34, 39, 42, 55, 62, 72, 83, 86, 88, 89, 90, 92	0.4702	0.002931
2	7, 13, 34, 39, 42, 55, 62, 72, 83, 86, 89, 90, 92	0.4699	0.002905

**Table 3:** Characteristics of the approximate Pareto-optimal solutions in Figure 10.

Solution number	Line sections open	Voltage deviation	Operated switches
1	84, 85, 86, 87, 88, 89, 90, 91, 92, 93, 94, 95, 96	0.0033805	0
2	34, 84, 85, 86, 87, 88, 89, 90, 91, 92, 93, 95, 96	0.0032245	1
3	34, 72, 84, 85, 86, 88, 89, 90, 91, 92, 93, 95, 96	0.0031690	2
4	7, 34, 62, 84, 86, 87, 88, 89, 90, 91, 92, 93, 95	0.0030271	3
5	7, 34, 62, 72, 84, 86, 88, 89, 90, 91, 92, 93, 95	0.0029678	4
6	7, 34, 62, 72, 83, 84, 86, 88, 89, 90, 92, 93, 95	0.0029392	5
7	7, 34, 55, 62, 72, 83, 86, 88, 89, 90, 92, 93, 95	0.0029166	6
8	7, 34, 39, 62, 72, 83, 84, 86, 88, 89, 90, 92, 95	0.0029314	6
9	7, 34, 39, 55, 62, 72, 83, 86, 88, 89, 90, 92, 95	0.0029087	7
10	7, 34, 39, 42, 55, 62, 72, 83, 86, 88, 89, 90, 92	0.0029046	8

**Table 4:** Characteristics of the Pareto-optimal solutions in Figure 11.

Solution number	Line sections open	Power losses (Mw)	Voltage deviation	Operated switches
1	84, 85, 86, 87, 88, 89, 90, 91, 92, 93, 94, 95, 96	0.5320	0.0033805	0
2	34, 84, 85, 86, 87, 88, 89, 90, 91, 92, 93, 95, 96	0.50957	0.0032245	1
3	7, 63, 84, 86, 87, 88, 89, 90, 91, 92, 93, 94, 95	0.49949	0.0032055	2
4	7, 62, 84, 86, 87, 88, 89, 90, 91, 92, 93, 94, 95	0.49992	0.0031927	2
5	7, 34, 84, 86, 87, 88, 89, 90, 91, 92, 93, 95, 96	0.48997	0.003276	2
6	34, 72, 84, 85, 86, 88, 89, 90, 91, 92, 93, 95, 96	0.50562	0.003169	2
7	7, 34, 62, 84, 86, 87, 88, 89, 90, 91, 92, 93, 95	0.4775	0.0030271	3
8	7, 34, 63, 84, 86, 87, 88, 89, 90, 91, 92, 93, 95	0.47707	0.0030406	3
9	7, 34, 62, 72, 84, 86, 88, 89, 90, 91, 92, 93, 95	0.47354	0.0029678	4
10	7, 34, 63, 72, 84, 86, 88, 89, 90, 91, 92, 93, 95	0.47311	0.0029816	4
11	7, 34, 63, 72, 83, 84, 86, 88, 89, 90, 92, 93, 95	0.47066	0.0029532	5
12	7, 34, 62, 72, 83, 84, 86, 88, 89, 90, 92, 93, 95	0.47109	0.0029392	5
13	7, 13, 34, 63, 72, 83, 84, 86, 89, 90, 92, 93, 95	0.47035	0.0029792	6
14	7, 34, 55, 62, 72, 83, 86, 88, 89, 90, 92, 93, 95	0.47046	0.0029166	6
15	7, 13, 34, 55, 62, 72, 83, 86, 89, 90, 92, 93, 95	0.47015	0.002943	7
16	7, 34, 39, 55, 62, 72, 83, 86, 88, 89, 90, 92, 95	0.47032	0.0029087	7
17	7, 34, 39, 42, 55, 62, 72, 83, 86, 88, 89, 90, 92	0.47019	0.0029046	8
18	7, 13, 34, 39, 55, 62, 72, 83, 86, 89, 90, 92, 95	0.47001	0.0029351	8
19	7, 13, 34, 39, 42, 55, 62, 72, 83, 86, 89, 90, 92	0.46988	0.0029311	9

**Table 5:** Cost associated with each line.

Nr	*w*1	*w*2	Nr	*w*1	*w*2
0	0.0000	0.0000	23	17.3314	34.806
1	31.6776	32.9922	24	72.6631	46.7469
2	65.3549	35.8537	25	68.0291	44.9918
3	44.9595	23.4323	26	51.6128	22.2112
4	16.1865	23.6162	27	16.5226	34.7425
5	21.971	38.0799	28	96.3964	17.0508
6	60.7701	42.5447	29	25.0725	16.6977
7	20.1846	39.823	30	37.0809	11.0776
8	15.6921	31.8862	31	74.4496	28.6943
9	12.3896	43.8891	32	66.3899	35.9456
10	38.2169	42.847	33	22.3423	31.9243
11	87.851	18.5533	34	85.3415	26.3923
12	27.4456	44.4433	35	75.702	30.5113
13	52.9695	34.3833	36	52.4566	15.5317
14	24.7319	23.5492	37	18.4093	32.6586
15	18.9167	28.3563	38	39.4639	39.5681
16	38.8376	36.9862	39	46.0833	28.0846
17	69.9022	36.481	40	54.0409	37.3085
18	56.6392	27.931	41	39.8044	20.8134
19	81.37	39.3791	42	69.8945	46.8211
20	53.6563	34.8405	43	41.2821	11.2217
21	47.2281	15.6442	44	39.8463	12.9746
22	99.8769	30.3881	45	90.4494	21.9358

## Appendix

Table 5 shows the costs w1 and w2 for each line section  in the minimum spanning tree problem of Section 3.
